# Evidence of Spatial Homogeneity in an Electromethanogenic Cathodic Microbial Community

**DOI:** 10.3389/fmicb.2019.01747

**Published:** 2019-07-31

**Authors:** Ala’a Ragab, Krishna P. Katuri, Muhammad Ali, Pascal E. Saikaly

**Affiliations:** Biological and Environmental Science and Engineering Division, Water Desalination and Reuse Center, King Abdullah University of Science and Technology, Thuwal, Saudi Arabia

**Keywords:** electromethanogenesis, spatial variability, CO_2_ reduction, biocathode, microbial community assembly

## Abstract

Microbial electrosynthesis (MES) has been gaining considerable interest as the next step in the evolution of microbial electrochemical technologies. Understanding the niche biocathode environment and microbial community is critical for further developing this technology as the biocathode is key to product formation and efficiency. MES is generally operated to enrich a specific functional group (e.g., methanogens or homoacetogens) from a mixed-culture inoculum. However, due to differences in H_2_ and CO_2_ availability across the cathode surface, competition and syntrophy may lead to overall variability and significant beta-diversity within and between replicate reactors, which can affect performance reproducibility. Therefore, this study aimed to investigate the distribution and potential spatial variability of the microbial communities in MES methanogenic biocathodes. Triplicate methanogenic biocathodes were enriched in microbial electrolysis cells for 5 months at an applied voltage of 0.7 V. They were then transferred to triplicate dual-chambered MES reactors and operated at -1.0 V vs. Ag/AgCl for six batches. At the end of the experiment, triplicate samples were taken at different positions (top, center, bottom) from each biocathode for a total of nine samples for total biomass protein analysis and 16S rRNA gene amplicon sequencing. Microbial community analyses showed that the biocathodes were highly enriched with methanogens, especially the hydrogenotrophic methanogen family *Methanobacteriaceae, Methanobacterium* sp., and the mixotrophic *Methanosarcina* sp., with an overall core community representing > 97% of sequence reads in all samples. There was no statistically significant spatial variability (*p >* 0.05) observed in the distribution of these communities within and between the reactors. These results suggest deterministic community assembly and indicate the reproducibility of electromethanogenic biocathode communities, with implications for larger-scale reactors.

## Introduction

Microbial electrochemical technologies (METs) have been heavily investigated for over a decade, specifically for applications in energy generation, waste reuse and resource recovery. Essentially, these are bioreactors with an anode and cathode where either one or both is biotic, allowing for oxidation (anode) and reduction (cathode) reactions. Electrode-assisted methanogenesis, or electromethanogenesis, refers to microbial electrochemical CO_2_ reduction to methane at the biocathode (biotic cathode). This process has been mainly investigated in microbial electrolysis cells (MECs) and, more recently, in microbial electrosynthesis (MES) (Blasco-Gómez et al., 2017). Applications for electromethanogenesis include bioelectrochemical power-to-gas, biogas upgrading, and wastewater treatment (Geppert et al., 2016; Blasco-Gómez et al., 2017).

In electromethanogenesis, CO_2_ is converted to methane using reducing equivalents generated from the cathode, either through direct uptake of electrons from the cathode surface (Rowe et al., 2019) or indirectly via H_2_. H_2_ is considered to be the main electron donor and can be produced abiotically through the hydrogen evolution reaction (HER) at the electrode surface at low cathode potentials (<-0.6 V vs. Ag/AgCl) or biotically by proton-reducers in mixed cultures such as sulfate-reducing bacteria (SRB) (Rosenbaum et al., 2011; van Eerten-Jansen et al., 2014; Batlle-Vilanova et al., 2015; Blasco-Gómez et al., 2017; Agostino and Rosenbaum, 2018). Formate, which can arise as an intermediate during CO_2_ reduction, can also serve as an indirect electron mediator (van Eerten-Jansen et al., 2014).

The microbial community at the biocathode of MES is less studied compared to MEC. In recent years, many MES studies have been done, focusing on methane production, as well as acetate and other volatile fatty acids (VFAs). These studies generally provide basic descriptions of the cathodic microbial community but with little analysis of the ecology and community assembly (Liu et al., 2016; Koch et al., 2018). There is generally a lack of in-depth analysis into why certain communities are present, their distribution and interactive networks for electromethanogenesis, except for some notable exceptions (Bretschger et al., 2015; Dykstra and Pavlostathis, 2017b). Understanding community assembly in MES is important as these systems rely entirely on their microbiome to function; a deeper understanding allows for microbial ecology-based engineering of efficient systems (Koch et al., 2018). Further, understanding community assembly dynamics in these systems enables the development of accurate models to predict reactor performance (Gadkari et al., 2018) and ensure predictable communities that function to achieve reproducibility and reliability for large scale applications (Kobayashi et al., 2013).

Community assembly refers to the species present in a community at a given space and time (Begon et al., 2006). There are two main theories on what drives microbial community assembly. The niche theory follows the assumption that certain microorganisms with specialized fitness are better suited to survive in certain environments or niches, and thus community assembly in these niches is driven by deterministic factors such as substrate availability and competition. On the other hand, the neutral theory assumes equal fitness amongst different microbial communities, with community assembly differences arising from stochastic factors such as birth/death and immigration (Hubbell, 2001). Although electroactive microbes do not belong to a unique ecological niche (Koch and Harnisch, 2016), METs create a highly selective niche environment for electroactive microbes. Electroactive microbes have an added fitness due to their ability to perform extracellular electron transfer to donate electrons to anodes in METs, which serves as the main deterministic driver of anodic community assembly, although stochastic assembly has been reported (Zhou et al., 2013; Cotterill et al., 2018). However, the cathode environment in MES creates two main selective factors: the ability to accept extracellular electrons using the cathode as the sole electron donor (through direct electron uptake or H_2_ due to proton reduction catalyzed by the cathode surface at lower potentials) and the capability of autotrophic growth as CO_2_ is the only externally added carbon source. Extracellular electron transfer capabilities (whether transfer to anode or uptake from cathode) have been demonstrated across a range of phyla, while autotrophic growth in the conditions set at the cathode in MES is thus far limited to mainly three phyla (Euryarchaeota, Firmicutes, and Proteobacteria) (Koch and Harnisch, 2016; Logan et al., 2019). Therefore, while there are studies into community assembly and spatial variability of bioanodes, those conclusions may not necessarily be applicable to MES biocathode community assembly.

No studies to date have investigated cathode spatial variability in methanogenic MES reactors. Spatial variability can arise due to a number of factors that affect microbial community assembly. While there is a bulk environment in these reactors, local microenvironments arise across the cathodes due to H_2_ and CO_2_ mass transfer limitations from the bulk solution into the biofilm, charge limitations across the biofilm as shown in other electrode-associated biofilms (Atci et al., 2016). Within the biofilm, further variations can occur in terms of differences in H_2_ gas bubble evolution along the cathode surface as a function of cathode roughness and H_2_ saturation (Vachaparambil and Einarsrud, 2018; Sapireddy et al., 2019). The pH gradients due to HER (Cai et al., 2018) affects CO_2_ solubility, and thus its availability, due to the shifts in the bicarbonate – carbonate equilibrium in response to pH change (Bajracharya et al., 2017). Collectively, these can potentially lead to spatial variability across the cathode biofilm, where certain sections may have higher or lower amounts of biomass and different types of microorganisms aggregating.

Understanding community spatial distribution and heterogeneity is important to ensure appropriate sampling strategies are undertaken. If there is significant spatial variability, wrong conclusions can be drawn depending on the number and position of samples. In larger scale electrodes, this spatial variability could be quite significant. Additionally, understanding spatial distribution can give insights on syntrophic or competitive relationships that occur. Mixed communities offer more robust and functionally redundant systems that are more suited to industrial applications due to their resiliency to possible operational fluctuations; understanding spatial variability of intact mature biofilms is important in predicting how these biofilms will behave under large scale applications. Therefore, the objective of this study was to evaluate the spatial distribution and variability across cathodic biofilms in an electromethanogenic MES system, as well as the reproducibility of biocathode community between biological replicates. We hypothesized that if deterministic factors are the dominant driving factor, then no significant difference in community composition (i.e., beta diversity) is expected between replicate reactors. In contrast, if neutral or stochastic factors prevail, then significant beta diversity would be observed.

## Materials and Methods

### MEC Set Up and Methanogenic Biocathode Enrichment

Triplicate single-chamber MEC reactors were prepared using 300 ml screw-capped borosilicate glass bottles, with a working volume of 280 ml. The caps and bottles were modified with appropriate ports to place the electrodes, gas collection bag (Calibrate, Inc., United States) and gas sampling port. The anodes, made of carbon fiber brush with a titanium core (4 cm × 2.5 cm, The Mill-Rose Company, United States), were cleaned by soaking in acetone overnight, washing with sterile deionized water and heat-treated at 450°C for 15 min. Carbon cloth cathodes were prepared with 160 cm^2^ (8 cm length × 10 cm width) geometric surface area, with titanium wire woven through as the current collector. The cathodes were cleaned by soaking in acetone overnight, washed with sterile deionized water and dried at room temperature. The anode and cathode were positioned vertically within the reactor, approximately 2 cm apart. Teflon tape and epoxy were applied on all the connections to ensure a proper seal. Reactors were inoculated with sludge from an anaerobic membrane bioreactor (10% v/v), mixed with a synthetic influent medium containing 10 mM sodium acetate as the carbon source and electron donor. The influent medium was prepared using a modified DSMZ Medium 826 (DSMZ, Leibniz, Germany) with the following composition (g/L): NH_4_Cl, 1.5; Na_2_HPO_4_, 0.6; KCl, 0.1; Na_2_HCO_3_, 2.5; CH_3_COONa, 0.82, and 10 ml trace minerals and vitamin solution each. To maintain anaerobic conditions, the media was sparged for 1 h using a N_2_:CO_2_ (80:20) gas mixture and then autoclaved. The sodium bicarbonate was sterile filtered into the media after autoclaving to maintain a pH of ∼7.5. The reactors were operated in fed-batch mode with an applied voltage of 0.7 V using an external power source (3645 A; Circuit Specialists, Inc., United States). A data logger (ADC 24, PicoLog, United Kingdom) was used to measure the voltage across an external resistor (R_ex_ = 10 Ω). A 10% decrease in voltage from the peak reading signaled the end of each batch for media replacement and sampling. This was approximately every 48 h. The reactors were enriched for a total of 5 months, after which the enriched methanogenic biocathodes were transferred to sterile triplicate double-chambered three-electrode MES reactors.

### MES Set Up and Operation

**FIGURE 1:**
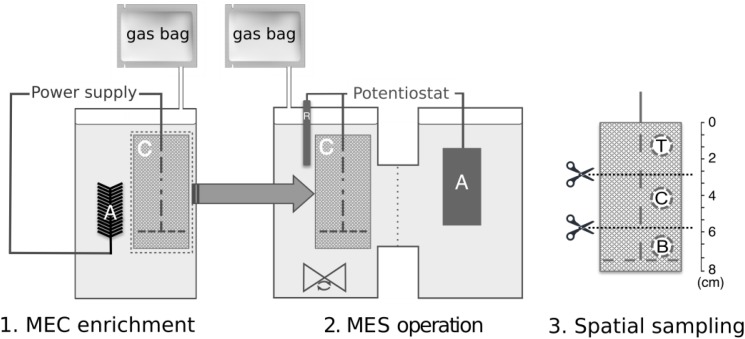
Experimental set-up and workflow, indicating (1) MEC enrichment phase followed by transfer of biocathode to (2) dual-chambered MES reactors, and (3) spatial sampling along the biocathode of MES at positions T, top; C, center; and B, bottom, for subsequent protein and DNA extraction and analysis. “A,” “C,” and “R” within the reactor schematic refer to anode, cathode, and reference electrode, respectively.

For the double-chambered MES reactors, the anodes were titanium sheets using titanium wires as the current collectors. A Nafion^®^ 117 cation exchange membrane (Sigma, United States) was used to separate the double chambers. Gas bags were attached to gas outlet ports to collect biogas produced during each batch for analysis. The same MEC enrichment media composition was used for the MES operation with the omission of sodium acetate, and continuous stirring. Therefore, the only carbon source was CO_2_ in the form of dissolved sodium bicarbonate for pH adjustment and 100% CO_2_ gas, which was continuously bubbled into the reactors at the beginning of each batch for 5 min and acted as a CO_2_ reservoir through passive gas diffusion from the gasbags into the reactor headspace. An Ag/AgCl reference electrode (BASi, United States) was inserted in the cathode chamber to maintain the set potential control. A VMP3 potentiostat (BioLogic, United States) was connected to the three-electrode system to chronoamperometrically maintain a cathode set potential of -1.0 V vs. Ag/AgCl. The reactors were batch-fed with each batch lasting 140 h. Once stable methane production was observed for three batches, the biocathodes were removed for microbial community analyses. To minimize disturbance to the biofilm, biocathode sampling was done only at the end of the experiment. Three 2 cm^2^ samples were cut using sterilized scissors from each biocathode at the top, center, and bottom positions (Figure 1) and suspended in 6 ml sterile media. These were vortexed for 1 min to detach the microbial cells from the cathode and stored at -80°C for subsequent protein analysis, DNA extraction and amplicon sequencing.

### Measurement and Analyses

Liquid and gas (H_2_, CH_4_, and CO_2_) samples were measured at the end of each batch cycle using chromatographic methods. Volatile fatty acids were detected at 210 nm using an Aminex HP-87H column (Bio-Rad, Hercules, CA, United States) with a UV-detector high performance liquid chromatography (HPLC; Shimadzu, Japan). The mobile phase was 0.005M H_2_SO_4_ at a flow rate of 0.55 ml/min. Samples were filtered through 0.2 um filters prior to analysis. The gas compositions in the reactor headspace and gas bag were analyzed using a gas chromatograph (Model# 8610C, SRI Instruments, United States) as previously described (Hari et al., 2017).

#### MEC Calculations

Current density *j* (mA/cm^2^) was calculated as:

$$I=\frac{V}{R}$$

where I was the current (mA) calculated from the recorded voltage (mV) across the resistor (1000 mΩ, R), divided by the geometric surface area of the cathode (160 cm^2^). Coulombic efficiency (CE%) was calculated as:

$$\mathrm{CE}=\frac{C_{t}}{C_{\mathrm{th}}}\times 100$$

where C_t_ is the total coulombs calculated by integrating the current over time (C_t_ = Σ *I* Δt, Δt is the cycle duration), C_th_ is the theoretical amount of coulombs available based on the acetate removed over the same amount of time, calculated as C_th_ = [*F b* (*C*_in_ – *C*_out_)]/*M*, where *F* is Faraday’s constant (96485 C/mol), *b* = 8 is the number of electrons produced per mole of acetate, *C*_in_ and *C*_out_ are the influent and effluent acetate concentrations and *M* = 82 is the molecular weight of acetate (Werner et al., 2016).

#### MES Calculations

The current density was calculated as the recorded current (mA) divided by the geometric surface area of the cathode (160 cm^2^). Coulombic efficiency (CE%) was calculated as the actual total coulombs C_t_ recovered as H_2_, CH_4_, formate, and acetate divided by the theoretical total coulombs C_th_ as recorded by the potentiostat software. Cathodic hydrogen and methane recoveries (r_catH2_ and r_catCH4_) were calculated by:

$$r_{\mathrm{catCH2}}=\frac{n_{\mathrm{H2}}}{n_{\mathrm{CE}}}$$

$$r_{\mathrm{catCH4}}=\frac{n_{\mathrm{CH4}}}{n_{\mathrm{CE}}}$$

where n_H2_ and n_CH4_ are the moles of the respective gas. n_CE_ is the total moles of gas possible based on the total coulombs C_t_, and is calculated by:

$$n_{\mathrm{CE}}=\frac{C_{t}}{\mathrm{bF}}$$

where b is the number of moles of electrons required for hydrogen production (2 mol e^-^) or methane production (8 mol e^-^) and F is Faraday’s constant (96,485 C/mol e^-^).

### Microbial Community Analyses

#### Protein Analysis

The total protein was measured based on the Lowry method (Lowry et al., 1951). The suspended samples described above (see section “MES Set Up and Operation”) were thawed at room temperature and the total protein was determined using the DC-protein assay kit (BIO-RAD Laboratories, Inc., United States) following the manufacturer’s instructions after being re-suspended in deionized (DI) water, with a series of graded Bovine Serum Albumin (BSA, Sigma Aldrich, United States) solutions as standards (0–0.5 μg/μl, *R*^2^> 0.97) (Bretschger et al., 2015; Dykstra and Pavlostathis, 2017a; Bian et al., 2018).

#### DNA Extraction, Library Preparation, Amplicon Sequencing, and Bioinformatic Processing

Genomic DNA was co-extracted with RNA from the carbon cloth and 150 μl of the cell suspension in which it was stored in using the PowerBiofilm RNA Isolation Kit (Qiagen, Germany) with a modified protocol using phenol:chloroform:isoamyl alcohol pH 6.5–8.0 (AMRESCO, Inc., United States) and bead beating lysing matrix E tubes (MP Biomedicals, New Zealand) instead of the original bead beating tubes. The extracted DNA concentration was measured using Qubit^®^dsDNA HS Assay Kit (Thermo Scientific, United States), according to the manufacturer’s instructions.

Amplicon libraries were prepared for the archaeal and bacterial 16S rRNA gene V3–V4 region using up to 10 ng of the extracted DNA, the forward primer Pro341F (5′-CCTACGGGNBGCASCAG-3′) and the reverse primer Pro805R (5′-GACTACNVGGGTATCTAATCC-3′) (Hari et al., 2016). Each PCR reaction (25 μL) contained dNTPs (100 μM of each), MgSO_4_ (1.5 mM), Platinum Taq DNA polymerase HF (0.5 U/reaction), Platinum High Fidelity buffer (1x) (Thermo Fisher Scientific, United States) and tailed primer mix (400 nM of each forward and reverse primer). The PCR amplification was conducted by an initial denaturation step at 95°C for 2 min, 35 cycles of amplification (95°C for 20 s, 50°C for 30 s, 72°C for 60 s) and a final elongation at 72°C for 5 min (Sapireddy et al., 2019). Duplicate PCR reactions were performed for each sample and the duplicates were pooled after PCR. The resulting amplicon libraries were purified using the standard protocol for Agencourt Ampure XP Beads (Beckman Coulter, United States) with a bead to sample ratio of 4:5. DNA concentrations were measured using the Qubit^®^dsDNA HS Assay Kit, followed by product size and purity validation with gel electrophoresis using Tapestation 2200 and D1000/High sensitivity D1000 screentapes (Agilent, United States).

Sequencing libraries were prepared from the purified amplicon libraries using a second PCR. Each PCR reaction (25 μL) contained PCRBIO HiFi buffer (1x), PCRBIO HiFi Polymerase (1 U/reaction) (PCRBiosystems, United Kingdom), adaptor mix (400 nM of each forward and reverse) and up to 10 ng of amplicon library template. The PCR amplification was conducted by an initial denaturation step at 95°C for 2 min, 8 cycles of amplification (95°C for 20 s, 55°C for 30 s, 72°C for 60 s) and a final elongation at 72°C for 5 min. The resulting sequencing libraries were purified as mentioned above using the Agencourt Ampure XP Beads. DNA concentration, product size and purity were measured as mentioned above. The purified sequencing libraries were pooled in equimolar concentrations and diluted to 2 nM. The samples were paired-end sequenced (2 bp × 300 bp) on a MiSeq using a MiSeq Reagent kit v3 (Illumina, United States) following the standard guidelines for preparing and loading samples on the MiSeq. > 10% PhiX control library was spiked in to overcome low complexity issues often observed with amplicon samples.

Forward and reverse reads were trimmed for quality using Trimmomatic v. 0.32 (Bolger et al., 2014) with the settings SLIDINGWINDOW:5:3 and MINLEN: 275. The trimmed forward and reverse reads were merged using FLASH v. 1.2.7 (Magoc and Salzberg, 2011) with the settings -m 10 -M 250. The trimmed reads were dereplicated and formatted for use in the UPARSE workflow (Edgar, 2013). The dereplicated reads were clustered, using the usearch v. 7.0.1090 -cluster_otus command with default settings. Operational taxonomic unit (OTU) abundances were estimated using the usearch v. 7.0.1090 -usearch_global command with -id 0.97 -maxaccepts 0 -maxrejects 0. Taxonomy was assigned using the RDP classifier (Wang et al., 2007) as implemented in the parallel_assign_taxonomy_rdp.py script in QIIME (Caporaso et al., 2010), using –confidence 0.8 and the MiDAS database v. 1.23 (Mcilroy et al., 2017), which is a curated database based on the SILVA database, release 123 (Quast et al., 2013). The results were analyzed in R v. 3.5.0 (R Core Team, 2017) through the RStudio using the ampvis2 package, which was also used to visualize the relative read abundance as a heatmap (Albertsen et al., 2015). The log abundance ratio was calculated by taking the log (base 10) of the ratio of the OTU relative abundance of organism X (OTU_X) to organism Y (OTU_Y) from within the same sample:

$$\log\;\mathrm{abundance}\;\mathrm{ratio}=\log[\mathrm{relative}\;{\mathrm{abundance}}_{\mathrm{OTU}\_X}/\mathrm{relative}\;{\mathrm{abundance}}_{\mathrm{OTU}\_Y}]$$

### Statistical Analyses

Statistical analyses were performed in RStudio using the base R, the ampvis2 package for alpha diversity and rank abundance of the core community (Andersen et al., 2018), and QIIME 1.9.1 for beta diversity analysis (Caporaso et al., 2010). The normality of data distribution was examined by the Shapiro–Wilk test. The two-tailed (independent) Student’s *t*-test was used to compare means between unpaired groups with an assumption of unequal variance between sample sets. The Mann–Whitney *U*-test was used to compare non-parametric variables between two groups. One-way analysis of variance (ANOVA) was used to compare parametric variables among three or more groups, and the Kruskal–Wallis test was used for non-parametric variables. Quantitative variables were expressed as the mean ± standard deviation or median and interquartile range, according to the sample distribution. *p*-Values less than 0.05 were considered to indicate statistical significance against the null hypothesis of no variance.

The beta-diversity dissimilarity analyses were done using the Bray–Curtis and Weighted UniFrac metrics with the beta_diversity.py script in QIIME. The Bray–Curtis dissimilarity is based on abundance, while Weighted UniFrac distance matrix calculates dissimilarity based on abundance and phylogeny. These results were visualized using non-metric multidimensional scaling (nMDS) with the ampvis2 R package. To assess the significance of the calculated beta-diversity dissimilarities, pairwise analyses of similarities (ANOSIM) based on 999 permutations and Adonis/permutational multivariate analysis of variance (PERMANOVA) based on 719 permutations were performed to compare each reactor and each sampling position groups using compare_categories.py for ANOSIM and Adonis, wrapping in the vegan R package (Oksanen et al., 2010). The ANOSIM R statistic is based on the difference of mean ranks between groups and within groups and ranges from 0 (no separation) to 1 (complete separation). The ANOSIM *R* statistic is based on the difference of mean ranks between groups and within groups and ranges from 0 (no separation) to 1 (complete separation). The Adonis/PERMANOVA pseudo-*F* statistic operates on ranked dissimilarity, comparing the total sum of squared dissimilarities between groups to the squared dissimilarity within groups. Larger *F*-ratios indicate greater group dissimilarity. Statistical significance is determined comparing the statistic values (*R* or *F* depending on the test) retrieved from multiple permutations of the test (Buttigieg and Ramette, 2014). The beta-diversity analyses were performed using raw reads, reads rarefied to 41,955, reads normalized by cumulative sum scaling (CSS), and reads normalized by DESeq2 to assess if different normalization methods would affect the statistical significance of the calculated dissimilarities. Rarefaction involves subsampling all samples to an even depth without replacement. CSS involves scaling only the relatively invariant counts across samples, to reduce the influence of larger count values in the same matrix column (Paulson et al., 2013). DESeq2 calculates a scaling factor for each OTU in each sample based on the median of the scaling factors of the mean across all samples. It assumes a Negative Binomial distribution and minimizes the influence of large count values on the values of other OTUs allowing for increased sensitivity for smaller datasets (Love et al., 2014). CSS and DESeq2 were implemented with the normalize_table.py script in QIIME.

### Nucleotide Sequence Accession Numbers

The 16S rRNA gene sequencing reads have been deposited in the National Center for Biotechnology Information (NCBI) under BioProject ID PRJNA541055 with Accession Nos. SAMN11571464–SAMN11571473.

## Results

### MES Performance

**FIGURE 2:**
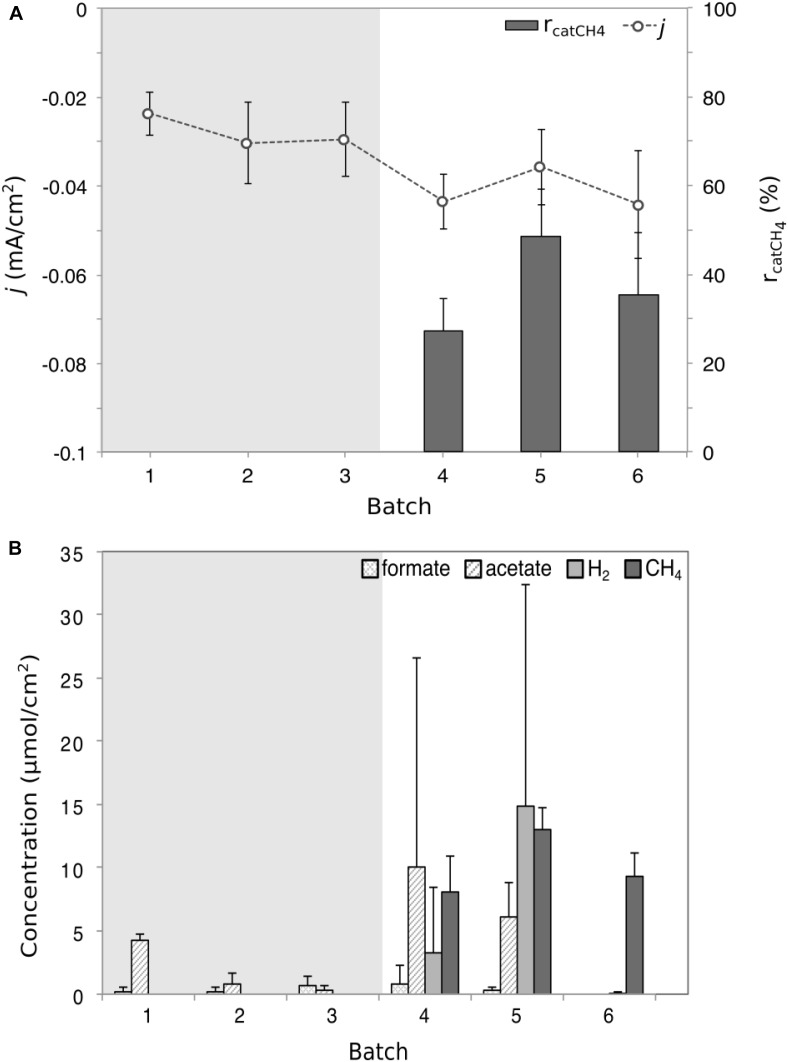
Performance and product formation plots for the microbial electrosynthesis (MES) reactor. **(A)** The recorded average current density *j* (dot and line plot) and the cathode recovery efficiency for CH_4_ (rcat_CH4_, bar chart) for the six MES batches. **(B)** The average concentrations of the four detected products in the form of formate, acetate, methane, and hydrogen gas. The shaded gray area indicates the period of time where gas analysis was not done; therefore no gas data were available and it was not possible to calculate r_catCH4_. Each product data point represents the average (triplicate reactors) recorded for each batch test. Current density was significantly variable (ANOVA, *F* = 5.1, *p* = 0.001), as was H_2_ concentration (Kruskal–Wallis, χ^2^ = 5.1, *p* = 0.02) and acetate concentration (Kruskal–Wallis, χ^2^ = 21.9, *p* < 0.001).

The reactors were operated for 5 months in MEC mode to enrich for methanogens, indicated by the detection of methane. The enriched methanogenic biocathodes were transferred to double-chambered MES reactors and operated in batch-fed mode for six batches (140 h batch-length) until stable methane production was observed in the last three consecutive batches (ANOVA, *F* = 4.2, *p* > 0.05, Supplementary Table S1). The MES cathodic current density (0.02–0.04 mA/cm^2^) was similar to that observed during the MEC operation (0.02–0.07 mA/cm^2^), with an overall increase in current consumption over time (Figure 2A and Supplementary Table S1). While there was little variability between reactors in each batch, there was a significant difference between batches (ANOVA, *F* = 5.1, *p* = 0.001), with the exception of methane production, which consistently averaged between 8 and 13 μmol/cm^2^ (Figure 2A). H_2_ production was more variable (Kruskal–Wallis, χ^2^ = 5.1, *p* = 0.02), reaching as high as 14 μmol/cm^2^ (Batch 5) and as low as 0.08 μmol/cm^2^ (Batch 6). The average HER rate was 0.04 μmol/cm^2^/h, compared to an abiotic HER rate of 6.3 μmol/cm^2^/h. The r_catH2_ (113 ± 7.5%, Supplementary Table S1) in the abiotic control reactor was much higher than for the biotic r_catH2_ (average of 4.9 ± 6.4%). Both electrode-assisted methanogenesis and acidogenesis occurred, as evidenced by the products detected at the end of each batch (methane, formate, and acetate) (Figure 2B). The VFA production was more significantly variable than gas production. Formate was detected in minimal amounts compared to the other products. Acetate levels varied significantly between batches (Kruskal–Wallis, χ^2^ = 21.9, *p* < 0.001), reaching a maximum of 10 μmol/cm^2^ to undetectable concentrations in Batch 6.

### Biomass Analysis and Alpha Diversity

**FIGURE 3:**
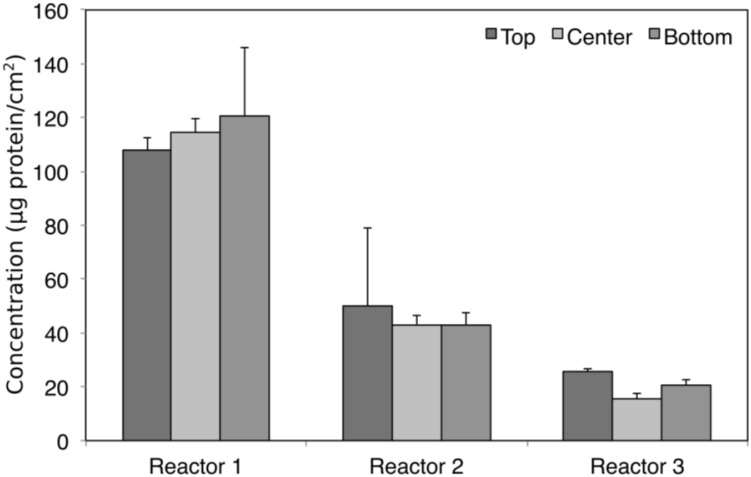
Biomass protein concentration at different sampling positions in each of the replicate reactors. No statistically significant differences were found for protein concentration between sampling positions (Kruskal–Wallis, χ^2^ = 0.047, df = 2, *p* > 0.05), although it varied significantly between reactors (Kruskal–Wallis, χ^2^ = 15.16, df = 2, *p* = 0.0005).

Triplicate samples (top, center, and bottom) were taken from each of the replicate biocathodes at the end of the experiment once stable performance was achieved (as determined by methane production) to quantify biomass and characterize microbial community diversity through amplicon sequencing. Total biomass did not vary significantly by position (Kruskal–Wallis, χ^2^ = 0.047, *p* > 0.05), although it varied significantly between the biological replicates (Kruskal–Wallis, χ^2^ = 15.16, *p* = 0.0005), with the highest total biomass measured in Reactor 1 (Figure 3).

**FIGURE 4:**
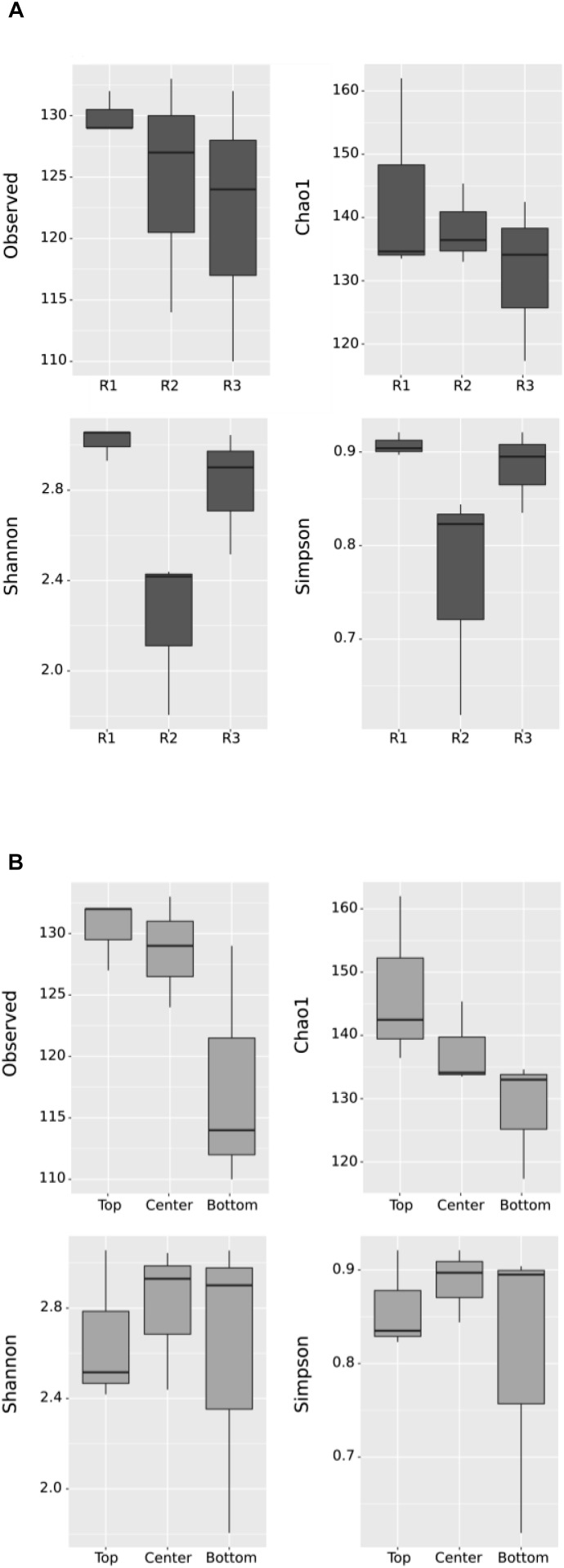
Box plot of alpha diversity using observed OTUs, Chao1, Shannon–Weaver and Simpson diversity indices by **(A)** Reactor and **(B)** Position. “R1,” “R2,” and “R3” refer to Reactor 1, Reactor 2, and Reactor 3. Each box represents the middle 50% of the data, while the middle quartile marks the mid-point. The lower quartile presents the 25% of scores that fall below the inter-quartile, while the upper quartile represents the 25% above the inter-quartile. No significant difference, except in the Shannon plot by Reactor, in **(A)** (ANOVA, *F* = 7.34, *p* = 0.0244).

Sequence reads after quality filtering ranged between 41,944 and 75,499, for a total of 473,048 reads which were resolved into 263 total observed OTUs. The sampling depth was sufficient to capture most of the species in the samples, as seen in the rarefaction curves (Supplementary Figure S1). Diversity in each sample was calculated based on the number of observed OTUs, Shannon–Weaver, Simpson’s Diversity and Chao1 richness estimator after rarefying to 41,944 reads. Shannon and Simpson diversity indices place more emphasis on abundant OTUs, whereas Chao1 takes into consideration rare OTUs. The results for the enriched biocathodes are presented based on reactor and position in Figure 4. Species richness (observed OTUs) was the highest in Reactor 1, with a median slightly greater than 130 observed OTUs, followed by Reactor 2 (median centered between 127 and 130 observed OTUs) and Reactor 3 (median centered between 123 and 125 OTUs). However, when considering Shannon–Weaver and Simpson diversity indices, Reactor 1 and 3 were most similar (Student’s *t*-test*, p* > 0.05), whilst Reactor 2 had the lowest alpha diversity, and was less evenly distributed as reflected in the rank abundance for the three reactors (Supplementary Figure S2), in which only four OTUs comprised more than 80% of the cumulative read abundance for Reactor 2 as compared to the other two reactors (11 OTUs for Reactor 1, 8 OTUs for Reactor 3). While alpha diversity was higher in Reactor 1 and 3 compared to Reactor 2, based on the abundance-based indices (i.e., Shannon–Weaver and Simpson diversity indices), there was less difference observed when considering the Chao1 richness and evenness index. No statistically significant difference was observed except when comparing the Shannon–Weaver diversity index between reactors (ANOVA, *F* = 7.34, *p* = 0.0244).

When comparing diversity based on position, the top samples had a higher diversity in terms of observed OTUs and Chao1 richness compared to bottom samples. This suggests some spatial heterogeneity across the cathode surface, with more richness observed in the top part of the cathode compared to the bottom regardless of the individual differences between reactors. However, when considering the dominant OTUs, no statistically significant difference was apparent in Shannon–Weaver and Simpson indices between the different sampling positions.

### Core Dominant OTUs

**FIGURE 5:**
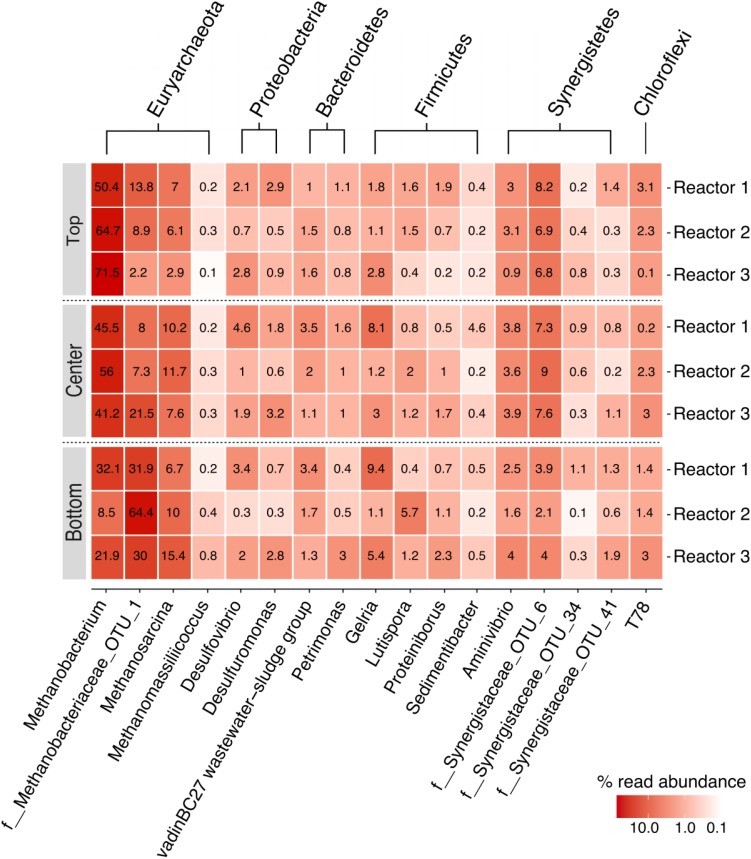
Heatmap of the relative read abundance (%) of the core community members for each of the three replicate reactors, rarefied to 41,944 reads. Taxonomic classification is indicated along the two x-axes; phylum-level classifications are shown along the secondary x-axis and genus level or lowest taxonomic classification (f: family) possible are shown along the primary x-axis.

In this study, the core dominant OTUs were defined as the OTUs present in all samples with a relative abundance ≥ 0.1%. The biocathodes were enriched with a core dominant OTUs representing 8% of total OTUs (21/263 total OTUs) and > 97% of total reads in all samples (Supplementary Figure S2). Of the 63 OTUs present at a relative abundance ≥ 0.1% in the initial anaerobic sludge inoculum (Supplementary Figure S3), only 10 were still represented at ≥ 0.1% in the final biocathode community for all samples. These 21 OTUs represented 17 core genera (or lowest taxonomic classification) belonging to the phyla Euryarchaeota (domain Archaea) and to Synergistetes, Bacteroidetes, Firmicutes, Proteobacteria, and Chloroflexi (domain Bacteria), as presented in a heatmap of relative abundance (Figure 5). The communities were dominated by the methanogenic archaeal communities and, to a lesser degree, a diverse group of bacteria. The biocathodes were highly enriched with hydrogenotrophic methanogens belonging to the family *Methanobacteriaceae* (five OTUs in total with no less than 41%, up to almost 70% relative abundance), and, to a lesser degree, *Methanosarcina* (one OTU) and *Methanomassiliicoccus* sp. (one OTU). The SRB, *Desulfovibrio* and *Desulfuromonas* sp., were relatively equally distributed across the cathode, with a relative abundance between 0.3 up to 4.6%.

**FIGURE 6:**
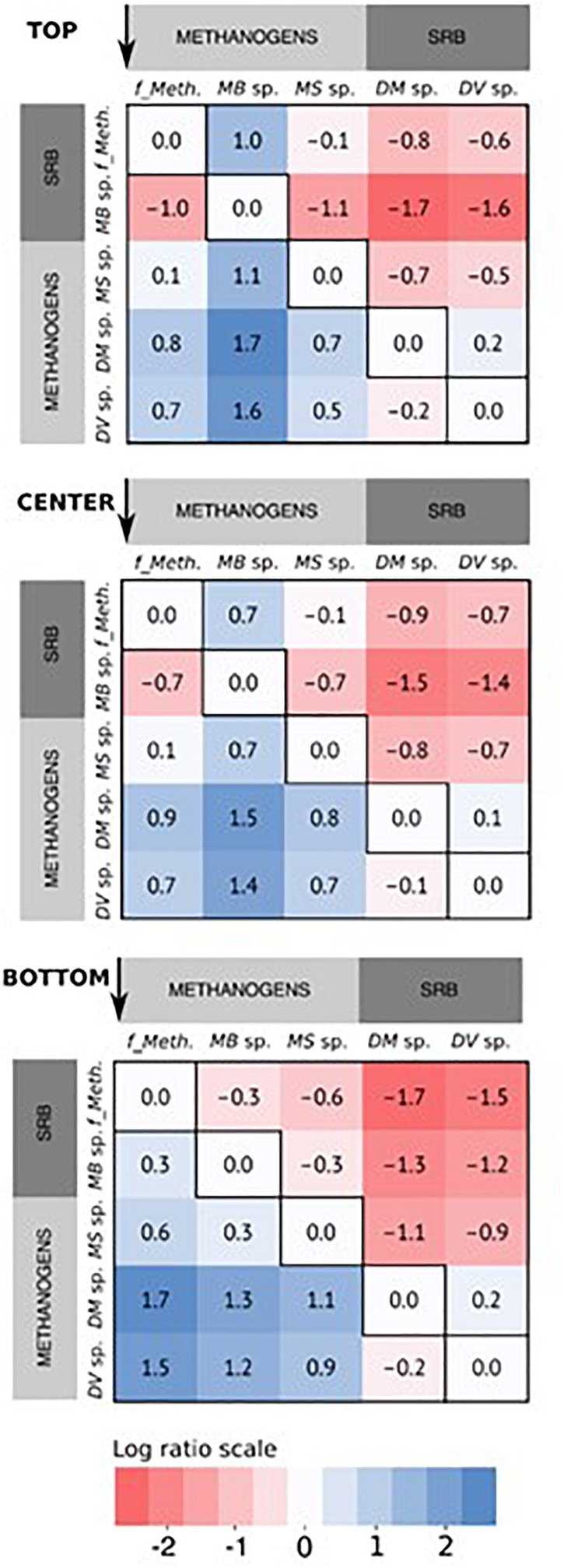
Heatmap of the log abundance ratio of the core community of methanogens and sulfate-reducing bacteria (SRB) arranged by sampling position, based on the average of the relative read abundance for the three replicates reactors. The ratios are presented as the ratio of the specific microbial community along the x-axis to that along the y-axis, in the direction indicated by the arrows for each figure. For the methanogens, “*f_Meth*.” is the family *Methanobacteriaceaea*, “*MB* sp.” is *Methanobacterium* sp., and “*MS* sp.” is *Methanosarcina* sp. For the SRBs, “*DM* sp.” is *Desulfuromonas* sp., and “*DV* sp.” is *Desulfovibrio* sp.

There appeared to be a preferential localization for some communities at the different positions on the cathode based on the relative abundance. This is more easily visualized by comparing log abundance ratios for the methanogenic and sulfate-reducing communities within each sample (Figure 6). With log ratios, every 0.33 represents a doubling in ratio, or every 1.0 represents a 10-fold increase. This means that for two OTUs with the same relative abundance, the log ratio would be zero, while if one OTU is two times more abundant, the log ratio would equal 0.33 and so on. While *Methanobacterium* sp. were the most relatively abundant community and were enriched across the cathode, they were more localized in the upper part of the cathode relative to the unclassified *Methanobacteriaceae* sp. (log ratio of 1) and *Methanosarcina* sp. (log ratio of 1.1), and almost two times less in the bottom part of the cathode compared to the *Methanobacteriaceae* sp. (log ratio -0.3). This seems to indicate a preferential localization for this community at the top of the cathode that decreased in the lower part of the cathode, in an inverse relationship to *Methanobacteriaceae* and *Methanosarcina* sp. While the log ratio decreased between *Methanobacterium* sp. and the two SRBs from the top to bottom, this appeared to be due to the decrease in relative abundance of *Methanobacterium* rather than a decrease in the SRB abundance, as evidenced by the log ratio comparing the two SRBs showing little difference regardless of cathode position (0.1–0.2). The other less abundant members of the core community also appeared to demonstrate less spatial variation; the results of their log ratio distributions are not shown in Figure 6 for simplicity.

### Beta Diversity

**FIGURE 7:**
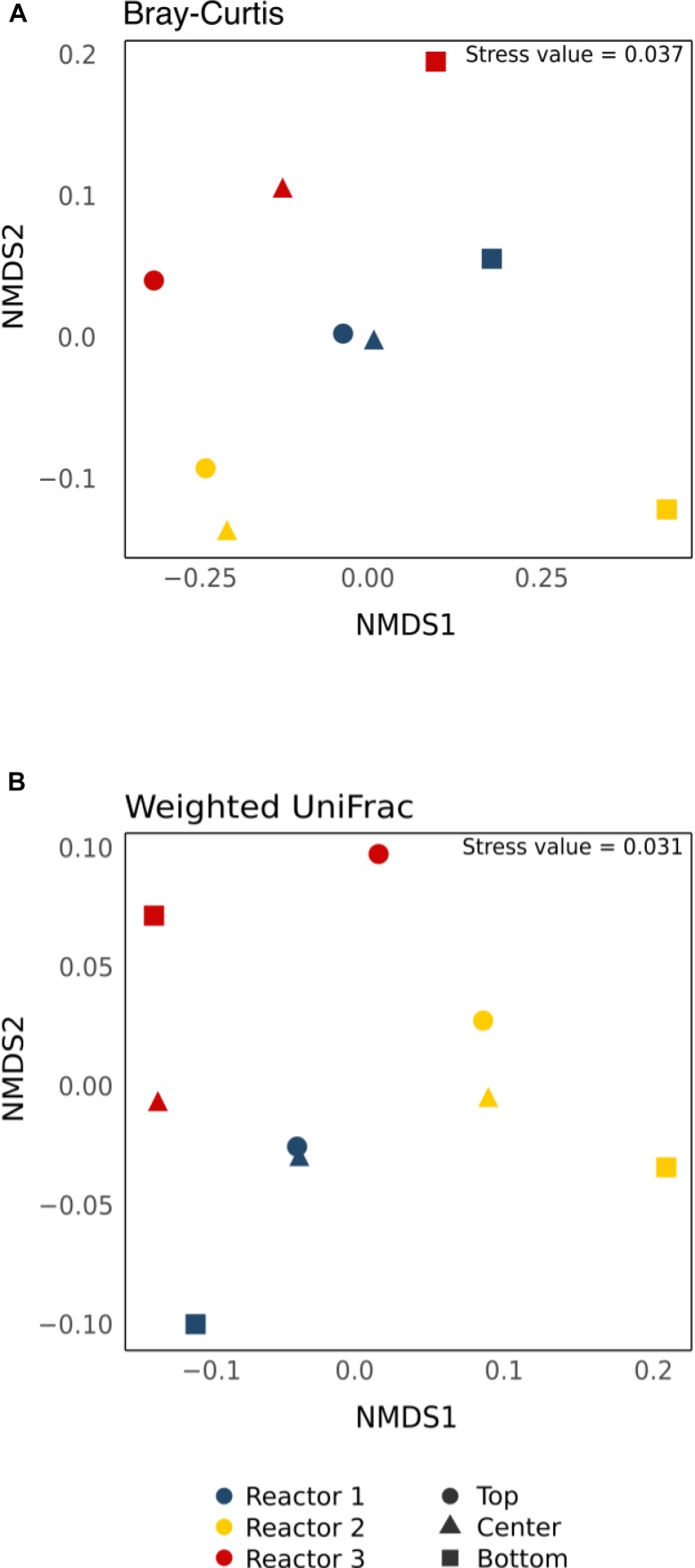
Non-metric multi-dimensional scaling (nMDS) plot of the core OTUs using **(A)** the non-phylogenetic Bray–Curtis dissimilarity matrix and **(B)** the phylogenetic-based Weighted UniFrac distance matrix. Colors indicate different reactors and shapes indicate different sampling positions.

Beta-diversity statistical analyses were done to determine the statistical significance of the observed spatial heterogeneity in relative abundance. The nMDS plots of the core community revealed that samples clustered more by reactor rather than by position regardless of the dissimilarity matrix used (Bray–Curtis or weighted UniFrac) (Figure 7). The data were normalized differently to see if beta-diversity results would be affected by normalization methods (McMurdie and Holmes, 2014; Weiss et al., 2017). Pairwise comparisons using ANOSIM or Adonis/PERMANOVA with raw OTU abundance data, data rarefied to 41,944 reads, data normalized by DESeq2 and CSS methods for the core OTUs and all the retrieved OTUs are shown in Supplementary Table S2. Pairwise comparisons revealed no significant differences in beta-diversity regardless of the normalization methods and whether core or all retrieved OTUs were used. In no case was there a significant difference from the null distribution (Supplementary Table S2, *p* > 0.05).

## Discussion

### Variations in Reactor Performance

Enrichment of the methanogenic biocathodes was done in single-chambered MECs prior to the dual-chambered MES operation to facilitate the growth of a mature methanogenic biocathode. HER rates are lower in dual-chambered reactors due to mass transfer limitations of protons across the cation exchange membrane separating the anode from the cathode. Several MES studies report an initial enrichment step prior to MES reactor transfer (Fu et al., 2015; Babanova et al., 2017; Zhen et al., 2018). While there was little variability between reactors within each batch during MES operation, there was a significant difference between batches in line with reported variability in methanogenic biocathode performance (Siegert et al., 2014; Bretschger et al., 2015; Babanova et al., 2017; Blasco-Gómez et al., 2017). Methane production was relatively consistent between batches compared to H_2_ production. It is important to note that r_catH2_ values (Supplementary Table S1) only reflect the average H_2_ detected at the end of each batch, and not the total H_2_ produced in the system. H_2_ is difficult to accurately quantify in such systems due to its role as an electron donor (biotic reaction) in the case of indirect electron transfer and because it can easily diffuse through the membrane and leak out from reactors and tubing (Ruiz et al., 2013; Dykstra and Pavlostathis, 2017a; Cotterill et al., 2018). This was further supported by the r_catH2_ (113 ± 7.5%, Supplementary Table S1) for the abiotic control reactor, which was significantly higher than for the biocathode (r_catH2_ of 4.9%). While most of this difference was due to H_2_-mediated methanogenesis, it is not clear how much of the evolved H_2_ was lost from the system. In either case, the H_2_ evolving from the cathode would not have been enough to account for the methane detected, since CO_2_ reduction to methane requires 4 moles of H_2_ for every mole of methane. The average amount of methane detected during the three MES batches was 1.6 mmol; an equivalent of ∼ 6.5 mmol H_2_ would be required to maintain the 1:4 stoichiometric ratio. However, the average abiotic H_2_ was only 0.89 mmol, indicating that abiotic HER was not the only source for reducing equivalents for methanogenesis, as has been previously reported (Dykstra and Pavlostathis, 2017a) which is discussed further in Section “Methanogen-Dominated Core Community.”

### Methanogen-Dominated Core Community

Absolute abundance values are not possible with just amplicon sequencing, and thus the data were only reported in terms of relative abundance. The relative abundance values may not fully capture the abundance of organisms due to the known limitations associated with amplicon sequencing, namely primer specificity, PCR, and 16S rRNA gene copy number variations between different species (Murray et al., 2015). Nevertheless, the methanogenic biocathodes were clearly highly enriched with a core community (>97% similarity across all the samples) that was made up of 60–80% hydrogenotrophic methanogens from the family *Methanobacteriaceae* (*Methanobacterium* sp. and an unclassified genus of *Methanobacteriaceae*) and *Methanosarcina* sp. (∼10%). Hydrogenotrophic methanogen communities are frequently reported to dominate methanogenic biocathodes, whether in MECs or MES, especially *Methanobacterium* sp. and *Methanobrevibacter* sp. (Siegert et al., 2015; Blasco-Gómez et al., 2017; Dykstra and Pavlostathis, 2017a). Members of the family *Methanobacteriaceae* are capable of reducing CO_2_ to CH_4_ in the presence of H_2_ (or formate) as an electron donor. H_2_-mediated methanogenesis is generally the most dominant pathway for methane generation in these systems as hydrogenotrophic methanogens do not contain cytochromes for direct electron uptake from the cathode (van Eerten-Jansen et al., 2014; Batlle-Vilanova et al., 2015; Blasco-Gómez et al., 2017), although *Methanospirillum hungatei* was recently reported to be capable of producing electrically conductive filaments (Walker et al., 2019). *Methanosarcina* spp. are metabolically versatile with mixotrophic growth; different species can produce CH_4_ through the three methanogenesis pathways (hydrogenotrophic, aceticlastic, and methylotrophic) (Kendall and Boone, 2006; Worm et al., 2010). While they contain cytochromes and are known to be involved in electroactivity (Rotaru et al., 2014; Rowe et al., 2019), they were present in lower abundance than the hydrogenotrophic methanogens, which is consistent with previous reports of methanogenic MES systems where *Methanobacteriaceae* spp. dominate the cathodic community (Blasco-Gómez et al., 2017).

Roughly 10% of the core community was represented by Proteobacteria. Members of the phylum Proteobacteria have been described as important members of methanogenic biocathodes, especially SRB like *Desulfovibrio* sp. and *Desulfuromonas* sp. which were enriched across the cathodes. *Desulfovibrio* spp. require an organic carbon source along with CO_2_ for growth due to their incomplete Krebs cycle (Pfennig, 1989), consuming carbohydrates and VFAs with H_2_ as an electron donor (Ariesyady et al., 2007) in the presence of sulfates. While SRB can outcompete hydrogenotrophic methanogens for H_2_ in the presence of sulfates due to their lower K_s_ and higher growth rates, under sulfate-limited conditions they instead establish a syntrophic relationship where SRB act as H_2_ producers and hydrogenotrophic methanogens as H_2_ consumers to maintain the thermodynamic favorability of the reaction (Muyzer and Stams, 2008; Werner et al., 2016). This was indeed the case in our reactors due to the presence of only trace amounts of sulfate in the media. Additionally, electrotrophy in SRB has been demonstrated, where they can directly accept electrons from the cathode and reduce protons to H_2_ since they contain hydrogenases (Aulenta et al., 2012; Agostino and Rosenbaum, 2018). Thus, the H_2_ evolution coupled with limited sulfate and acetate favored the growth of hydrogenotrophic methanogens, especially the observed *Methanobacterium* sp. and the other unclassified member of the *Methanobacteriaceae* family.

The remainder of the core community was made up of a diverse group of fermenters of the phyla Bacteroidetes, Synergistetes, Firmicutes, and Chloroflexi. Since no external organic carbon source was added, their presence was probably due to endogenous decay of the biofilm and amino acid fermentation, as has been previously reported (Dykstra and Pavlostathis, 2017b). These fermenters can produce acetate, H_2_, and CO_2_ as end products of their fermentation, and they are discussed further in the Supplementary Discussion. Although not part of the core dominant community (not present at a relative abundance of 0.1% or higher in all samples), a variety of genera capable of aerobic growth were also enriched at relative abundances > 0.1%. These included *Aquamicrobium* sp., *Thiobacillus* and the family *Comamonadaceae*. Aerobic microorganisms have been previously reported in other anaerobic bioreactors, including microbial fuel cells (Shehab et al., 2013), where it is expected that they persist by consuming any intruding oxygen in the system, thus aiding in maintaining an anaerobic environment. There may have been oxygen intrusion through the cation-exchange membrane separating the two chambers from the water-splitting abiotic anode (Chae et al., 2008; Sethuraman et al., 2009). The diversity of the microbiomes in the replicate reactors can help with stability and adaptability in the face of such destabilizing/unfavorable conditions.

### Log Ratio Abundance Highlights Preferential Spatial Localization of Hydrogenotrophic Methanogens

Although the hydrogenotrophic methanogens were relatively evenly distributed across the cathode, some spatial segregation was apparent for *Methanobacterium* sp. (four OTUs) and the unclassified *Methanobacteriaceae* sp. (one OTU) (Figure 5). *Methanobacterium* sp. were more highly abundant in the top samples versus the bottom, in a somewhat inverse relation to the *Methanobacteriaceae* sp.; this is more evident when comparing the log ratio abundance between the two communities (Figure 6).

Considering the broad range of species that belong to the family *Methanobacteriaceae*, the results suggested that these were two different spatially segregated hydrogenotrophic methanogen groups, possibly due to differences in their H_2_ utilization and growth kinetics. Different local microenvironments or niches may have developed at the top compared to the bottom of the cathode, resulting in spatial segregation of hydrogenotrophic methanogen communities. The H_2_ that evolves at the cathode does not reach an equilibrium state between the headspace and dissolved H_2_ due to its low solubility and density (Werner et al., 2016), and the dynamics between H_2_ production and microbial consumption rates across the cathode (Dykstra and Pavlostathis, 2017a). In this study, the rate of H_2_ production was a function of the abiotic HER and biotic H_2_ evolution by SRB and endogenous decay. The rate of H_2_ consumption is a function of the maximum H_2_ utilization rates (v_max_) and maximum specific growth rates (μ_max_) of the hydrogenotrophic methanogens (Conrad, 1999). The physical proximity of the top part of the cathode to the headspace, which has an abundance of H_2_ relative to the solution, may have resulted in relatively higher H_2_ availability in the compared to the bottom part of the cathode. Since the *Methanobacterium* sp. preferentially aggregated in the top, it may be inferred that this genus had a lower affinity to H_2_ (higher half-saturation constant, K_s_). The unclassified *Methanobacteriaceae* sp. may have had higher affinity to H_2_ (lower K_s_) allowing it to be more competitive in an environment with lower H_2_ availability, i.e., the bottom of the cathode. Higher versus lower HER rates have been shown to result in the dominance of different *Methanobacteriaceae* spp. in methanogenic biocathodes (Werner et al., 2016). In the case of similar H_2_ affinity, competition would be based on μ_max_ (Archer and Powell, 1985). Therefore, it is probable that H_2_ affinity and maximum specific growth rates differed between the two communities. It is not possible to determine exactly the species-level taxonomic classification with 16S rRNA amplicon sequencing, so no comparison of exact growth kinetics (i.e., K_s_ and μ_max_) can be made for the different species.

The distribution in relative abundance of the *Methanosarcina* sp. was more uniform across the cathode. As previously stated, they are capable of methanogenesis by using acetate, cathode, or H_2_ as an electron donor (Rotaru et al., 2014; Rowe et al., 2019). *Methanosarcina* sp. have a reported acetate threshold between 0.2 and 1.2 mM (Jetten et al., 1990). The maximum acetate concentration measured in the reactors was 1.6 mM, which is above the minimum threshold, although it varied between batches to below 0.2 mM. It should be noted that the acetate concentration was measured at the end of the batch and it is possible that the concentrations of acetate were higher during the batch and decreased with time. H_2_-driven methanogenesis would have led to direct competition between *Methanosarcina* sp. and other hydrogenotrophic methanogens, which have a lower K_s_ (H_2_) and thus a higher affinity to H_2_. A direct electron transfer mechanism by *Methanosarcina* sp. would not have involved competition for anything other than physical space to enable direct interaction with the cathode (along with H^+^ and CO_2_). It is possible that *Methanosarcina* sp. grew using a combination of acetate or direct electron transfer relatively independent of the H_2_ availability, leading to their uniform distribution across the cathode.

A similar uniformity of distribution was observed when comparing the abundances of the sulfate-reducing *Desulfovibrio* sp. and *Desulfuromonas* sp. As with the *Methanosarcina* sp., direct electron transfer for H^+^ reduction to H_2_ by the SRB would lead to uniformity across the cathode since no concentration gradients occur in terms of physical location. While the SRB abundance was relatively stable across the cathode in relation to each other (log ratio of 0.1–0.2, Figure 6), they were generally more highly abundant in the bottom part of the cathode as compared to the top of the cathode in relation to the *Methanobacterium* sp. This was due to the decrease in *Methanobacterium* sp. abundance in the bottom of the cathode. While *Methanobacterium* sp. abundance was overall lower in the bottom of the cathode, higher abundance was observed (>21%) in Reactor 1 and 3 samples with concurrently higher abundances of the SRB (>4%) compared to Reactor 2 (8.5% *Methanobacterium*), which had a lower abundance of SRB (0.6%) (Figure 5). This may suggest that *Methanobacterium* sp. had a stronger reliance on their syntrophic relationship with the SRB due to the lower H_2_ availability at the bottom.

**FIGURE 8:**
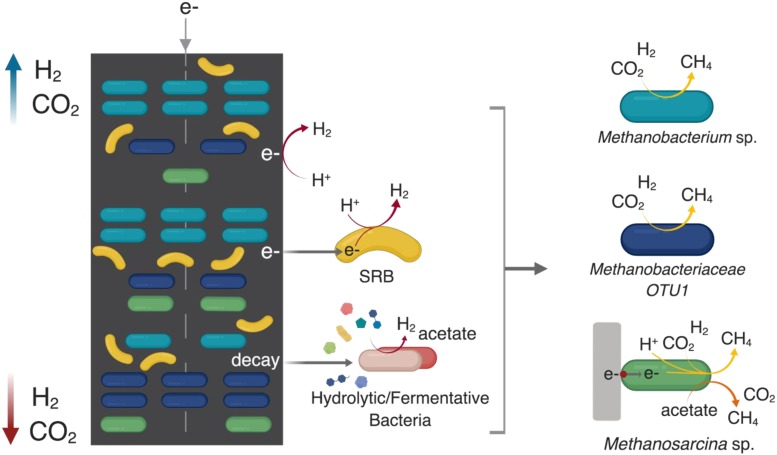
A hypothetical schematic developed based on the results of this study describing the spatial distribution of the key core community members along the cathode of methanogenic MES system where the cathode is the sole electron donor (direct or indirect via H_2_) and CO_2_ is the sole carbon source. ““SRB” refers to sulfate-reducing bacteria.” H_2_ can evolve directly from the surface of the cathode due to the reduction of H^+^ at < –0.6 V vs. Ag/AgCl. Endogenous decay within the cathodic biofilm can act as a source of complex substrates for various hydrolytic/fermentative bacterial communities (which were relatively equally distributed across the cathode) to produce intermediates (such as acetate, H_2_, and CO_2_) that are utilized by the different methanogens. H_2_ and CO_2_ partial pressure is higher in the headspace; thus they are relatively more available to the top part of the cathode (blue arrow), which is in closer proximity to the headspace compared to the bottom part (red arrow). SRB such as *Desulfovibrio* sp. can use the cathode as an electron donor to reduce protons available in the media due to the water-splitting reaction at the anode. In the absence of sulfates (or under sulfate-limited conditions), their syntrophic partnership with hydrogenotrophic methanogens maintains the thermodynamic favorability of SRB-driven H_2_ evolution where the methanogens consume H_2_ for CO_2_ reduction to methane. Generally, SRB partner with hydrogenotrophic methanogens of the family *Methanobacteriaceae*, of which *Methanobacterium* sp. are frequently described as dominant methanogens in methanogenic METs. The SRB-methanogen partnership is beneficial for hydrogenotrophic methanogens and they co-aggregated with *Methanobacterium* sp. at the bottom of the cathode. The metabolic versatility of *Methanosarcina* sp. to use the cathode as an electron donor, as well as produce methane via both the hydrogenotrophic and aceticlastic pathways allows them to be relatively evenly distributed throughout the cathode. The schematic was created using BioRender.com.

Figure 8 presents a hypothetical schematic describing these apparent spatial distribution trends of the core community members that are central to the functional performance of methanogenic MES in terms of current consumption, hydrogen production and methane production. Overall, it seemed the differences in the local segregation of the hydrogenotrophic methanogens could be mainly due to the difference in micro-scale H_2_ and CO_2_ availability as well as their growth kinetics (i.e., K_s_ and μ_max_).

### No Significant Beta-Diversity Within and Between Replicate Biocathodes

Despite the observed differences in relative abundance distribution, no statistically significant variance in beta-diversity was observed within and amongst the triplicate biocathodes, suggesting a deterministic-driven assembly of the cathodic microbial community. Seeded with the same inoculum, the microbial community at the cathode of replicate reactors converged to the same core community. This convergence to a core community of 21 OTUs supports a deterministic community assembly as has been shown in bioanodes and in anaerobic digestion (Dennis et al., 2013; Peces et al., 2018). Acting as the sole electron donor, the cathode creates a highly selective stress for chemolithoautotrophs capable of growth via direct electron transfer mechanisms or with H_2_ as an electron donor that clearly shapes the cathodic community, driving it toward a core community dominated by hydrogenotrophic methanogens (five OTUs). These results are promising, as they support the reproducibility of methanogenic MES biocathodic communities and their functional redundancy (i.e., different species that can perform the same function) which is important when considering larger scale applications subjected to operational fluctuations. Functional redundancy can help maintain the overall performance of the cathode, since differences in the cathode local micro-environments can arise in terms of HER variability that can occur due to cathode materials and pH gradients, H_2_ availability throughout the thickness of the biofilm, and syntrophic relationships that contribute to substrate (H_2_ and CO_2_) availability. The results highlight the importance of sufficient and appropriate sampling for microbial community analyses. Local variabilities in abundance can affect the conclusions drawn regarding factors shaping the community and the dominance of certain communities. Triplicate samples from multiple points across the cathode are the minimum needed for statistical analyses to determine whether observed variabilities are significant. However, many MEC and MES do not report their results in the framework of statistically relevant differences. It should be noted that amplicon DNA sequencing technique does not differentiate active from non-active members in the microbial community. Methods such as reverse-transcribed rRNA, can be applied in future studies, for identifying active populations to gain a deeper understanding of the functionally relevant interactions between communities.

## Conclusion

This study presents insights into the microbial community assembly, spatial distribution and homogeneity of electromethanogenic biocathodes. Our data showed that while the functional performance of these bioreactors may vary, it is unlikely to be due to differences in the overall communities present as deterministic assembly led to the development of a specific core community responsible for the majority of CO_2_ conversion to CH_4_ via different syntrophic relationships. Even though local community segregation may occur due to the differences in H_2_ utilization and competitive relationships, this did not result in any statistically significant overall beta diversity within the cathodes or between reactors. This information is relevant to understanding cathode community assembly in METs, especially those conducted with CO_2_ as the sole electron donor. For METs applied to wastewater treatment, where more complex organic substrates are available, obviously there would be differences in the core community assembly with a higher abundance of fermenters and heterotrophic growth due to the presence of larger amounts of fermentable substances, and stochastic community assembly may be stronger with continuous-operation reactors due to a regular immigrant influx. Additionally, this study highlights the importance of sufficient sampling for statistical analyses purposes that allow for more in-depth and meaningful investigations of sequencing data generated from the many MET studies carried out.

## Data Availability

The datasets generated for this study can be found in the 16S rRNA gene sequencing reads have been deposited in the National Center for Biotechnology Information (NCBI) under BioProject ID PRJNA541055 with Accession Nos. SRR9017425–SRR9017429.

## Author Contributions

AR, KK, and PS designed the experiments. AR performed the experiments, analyzed the data, and wrote the manuscript. MA provided guidance on the statistical analyses. AR, KK, MA, and PS contributed to the discussion and editing of the manuscript.

## Conflict of Interest Statement

The authors declare that the research was conducted in the absence of any commercial or financial relationships that could be construed as a potential conflict of interest.
