# Supplementary material for: Evidence of Spatial Homogeneity in an Electromethanogenic Cathodic Microbial Community
Source: Front Microbiol. 2019 Jul 31;10:1747. doi: 10.3389/fmicb.2019.01747 (PMC6685142; doi:10.3389/fmicb.2019.01747)
Supplement: Supplementary file 1 [file Data_Sheet_1.PDF]

Supplementary Material

Evidence of spatial homogeneity in an electromethanogenic cathodic microbial community

Ala'a Ragab[1], Krishna P. Katuri[1], Muhammad Ali[1], Pascal E. Saikaly[1*]

[1] Biological and Environmental Science and Engineering Division, Water Desalination and Reuse Center, King Abdullah University of Science and Technology (KAUST), Saudi Arabia

*** Correspondence:**
Pascal E. Saikaly
pascal.saikaly@kaust.edu.sa

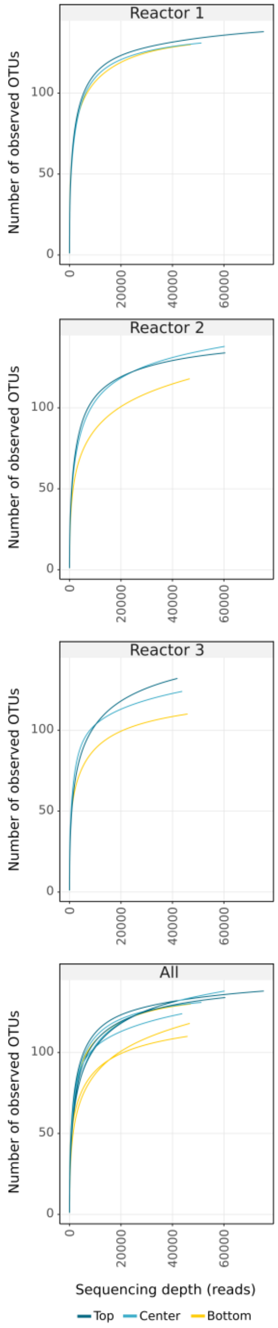

Figure S1. Rarefaction curves for all retrieved reads for the 263 operational taxonomic units (OTUs) for all samples faceted by reactors and combined together. Data points are colored by sampling position (top, center, bottom).

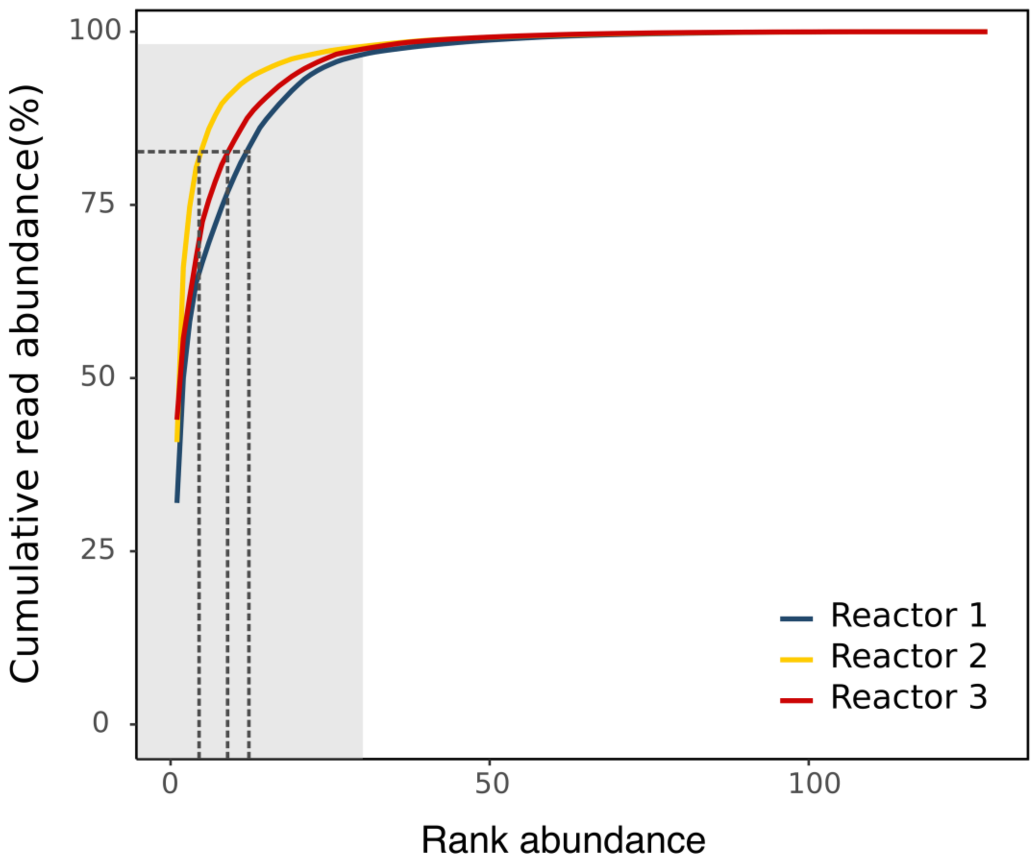

Figure S2. Rank abundance plot of cumulative reads (%) for all samples, grouped by reactor, after rarefaction to 41,955 reads. The dashed grey lines denote the highest ranked number of OTUs representing more than 80% of the cumulative read abundance. The shaded area highlights the number of OTUs representing more than 97% of the cumulative read abundance for all samples.

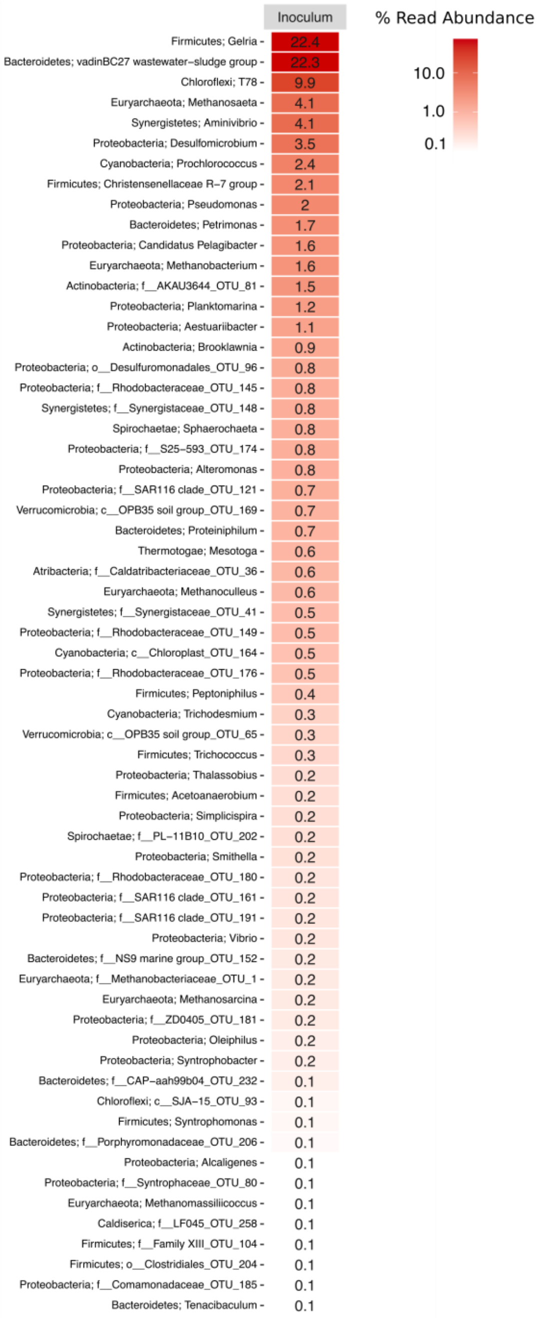

Figure S3. Heatmap of the initial inoculum community showing the 63 OTUs with relative read abundance ≥ 0.1%. Taxonomic classifications are shown as: phylum; genus or lowest taxonomic classification (c: class, o: order and f: family) possible.

Table S1. Conversion efficiencies for **(A)** microbial electrolysis cell (MEC) and **(B)** microbial electrosynthesis (MES) reactors, showing the overall Coulombic efficiency (CE%), and the proportion of the total coulombs available at the cathode captured as H_2 and CH_4, reported as cathode conversion efficiency, r_{catH2} and r_{catCH4}. One-way ANOVA (parametric data) and Kruskal-Wallis (non-parametric data) results all returned $p > 0.05$ except for H_2 concentration in MES (Kruskal-Wallis, $\chi^2 = 5.1$, $p = 0.02$). Each data point represents the average of the triplicate reactors. The average results of the abiotic reactors are presented in Table S1(B).

			MES			
**Batch**	**Current density (mA/cm²)**	**H_2 (mmol)**	**CH_4 (mmol)**	**CE%**	**r_{catH2} (%)**	**r_{catCH4} (%)**
**1**	–0.023 ± 0.005	–	–	–	–	–
**2**	–0.030 ± 0.009	–	–	–	–	–
**3**	–0.030 ± 0.008	–	–	–	–	–
**4**	–0.044 ± 0.006	0.52 ± 0.8	1.29 ± 0.45	65.1 ± 49.8	2.53 ± 4.1	27.18 ± 7.4
**5**	–0.036 ± 0.008	2.37 ± 2.8	2.09 ± 0.28	83.3 ± 17.0	12.14 ± 13.5	47.15 ± 9.8
**6**	–0.044 ± 0.012	0.01 ± 0.02	1.49 ± 0.29	35.5 ± 14.0	0.07 ± 0.08	34.14 ± 10.5
		**average**		**61.3 ± 24.1**	**4.9 ± 6.4**	**36.2 ± 10.1**
**abiotic**	–0.002 ± 0.0004	0.89 ± 0.3	–	113.0 ± 7.5	113.0 ± 7.5	–

Table S2. ANOSIM and ADONIS analyses results for pairwise comparisons of β-diversity for **(A)** all OTUs and **(B)** core community OTUs, using the Bray-Curtis dissimilarity and Weighted UniFrac distance metrics. In the "pairwise comparison" column, "R" refers to reactor, and "T", "C", "B" refer to the top, center, and bottom sampling positions, respectively. "Raw" refers to raw read counts, "rarefied" indicates rarefaction to 41,944 reads, "DESeq2" indicates normalization by the DESeq2 method, and "CSS" indicates normalization by cumulative sum-scaling (CSS) method. ANOSIM R values are based on 999 permutations and ADONIS F values are based on 719 permutations.

(A)

		**BRAY-CURTIS**				**WEIGHTED UNIFRAC**			
		**ANOSIM**		**ADONIS**		**ANOSIM**		**ADONIS**	
	Pairwise comparison	**R**	***p-value***	**F**	***p-value***	**R**	***p-value***	**F**	***p-value***
**RAW**	**R1 vs. R2**	0.33	0.20	1.41	0.30	1.00	0.09	0.09	0.10
	**R1 vs. R3**	0.52	0.12	2.98	0.10	0.07	0.43	1.42	0.40
	**R2 vs. R3**	0.30	0.20	1.70	0.20	0.52	0.10	4.36	0.10
	**T vs. C**	–0.22	0.71	0.51	0.80	0.04	0.29	1.11	0.20
	**T vs. B**	0.67	0.10	3.73	0.10	0.11	0.20	1.26	0.30
	**C vs. B**	0.30	0.11	2.54	0.10	-0.26	0.90	0.47	0.70
**RAREFIED**	**R1 vs. R2**	0.74	0.11	4.39	0.10	0.74	0.11	3.61	0.10
	**R1 vs. R3**	1.00	0.11	7.51	0.10	0.96	0.10	6.77	0.10
	**R2 vs. R3**	1.00	0.10	6.08	0.10	1.00	0.10	8.75	0.10
	**T vs. C**	–0.26	0.70	0.38	0.70	–0.37	1.00	0.38	0.70
	**T vs. B**	0.11	0.51	1.15	0.50	–0.07	0.61	0.76	0.50
	**C vs. B**	–0.07	0.58	0.79	0.70	–0.26	0.91	0.59	0.70
**DESEQ2**	**R1 vs. R2**	0.74	0.11	4.39	0.10	0.74	0.11	3.61	0.10
	**R1 vs. R3**	1.00	0.11	7.51	0.10	0.96	0.10	6.77	0.10
	**R2 vs. R3**	1.00	0.10	6.08	0.10	1.00	0.10	8.75	0.10
	**T vs. C**	–0.26	0.70	0.38	0.70	–0.37	1.00	0.38	0.70
	**T vs. B**	0.11	0.51	1.15	0.50	–0.07	0.61	0.76	0.50
	**C vs. B**	–0.07	0.58	0.79	0.70	–0.26	0.91	0.59	0.70
**CSS**	**R1 vs. R2**	0.85	0.09	4.00	0.10	0.89	0.10	4.85	0.10
	**R1 vs. R3**	1.00	0.10	6.83	0.10	1.00	0.10	8.08	0.10
	**R2 vs. R3**	1.00	0.08	6.40	0.10	1.00	0.10	11.16	0.10
	**T vs. C**	–0.22	0.70	0.40	0.70	–0.26	0.70	0.38	0.70
	**T vs. B**	–0.04	0.71	1.07	0.50	–0.11	0.72	0.70	0.50
	**C vs. B**	–0.15	0.68	0.66	0.70	–0.30	0.80	0.38	0.80

(B)

	Pairwise comparison	BRAY-CURTIS				WEIGHTED UNIFRAC			
		ANOSIM		ADONIS		ANOSIM		ADONIS	
		R	*p-value*	F	*p-value*	R	*p-value*	F	*p-value*
**RAW**	**R1 vs. R2**	0.26	0.20	1.38	0.30	0.93	0.10	9.66	0.10
	**R1 vs. R3**	0.48	0.10	2.81	0.10	0.04	0.42	1.24	0.40
	**R2 vs. R3**	0.26	0.21	1.62	0.20	0.52	0.10	4.26	0.10
	**T vs. C**	–0.22	0.70	0.52	0.80	0.04	0.29	1.17	0.20
	**T vs. B**	0.70	0.09	3.87	0.10	0.11	0.19	1.24	0.30
	**C vs. B**	0.33	0.09	2.65	0.10	–0.19	0.89	0.50	0.70
**DESEQ2**	**R1 vs. R2**	0.81	0.08	4.56	0.10	0.70	0.10	3.33	0.10
	**R1 vs. R3**	1.00	0.10	6.61	0.10	0.93	0.11	5.29	0.10
	**R2 vs. R3**	1.00	0.11	5.52	0.10	1.00	0.10	7.13	0.10
	**T vs. C**	–0.11	0.69	0.48	0.70	–0.19	0.71	0.53	0.70
	**T vs. B**	0.11	0.51	1.25	0.40	–0.07	0.63	0.85	0.60
	**C vs. B**	–0.07	0.69	0.81	0.70	–0.19	0.79	0.76	0.60
**CSS**	**R1 vs. R2**	0.70	0.10	4.15	0.10	0.74	0.09	4.29	0.10
	**R1 vs. R3**	1.00	0.10	6.86	0.10	0.74	0.09	5.80	0.10
	**R2 vs. R3**	1.00	0.09	5.60	0.10	0.93	0.09	6.56	0.10
	**T vs. C**	–0.15	0.69	0.44	0.70	–0.22	0.71	0.43	0.70
	**T vs. B**	0.15	0.49	0.01	0.40	–0.04	0.70	0.93	0.60
	**C vs. B**	0.04	0.54	0.81	0.60	–0.19	0.73	0.64	0.70

Supplementary Discussion

1. Fermenters

Roughly 25% of the core community was made up of a diverse group of fermenters of the phyla Bacteroidetes, Synergistetes, Firmicutes and Chloroflexi. Since no external organic carbon source was added in the media, their presence was probably due to endogenous decay of the biofilm and amino acid fermentation. Four Synergistetes OTUs were present, of which only *Aminivibrio* was classified at the genus-level. *Aminivibrio* spp. ferment amino acids and some organic acids to produce acetate, H_2 and CO_2 as end products (Honda et al., 2013). Synergistetes spp. in general are strictly anaerobic fermenters, with species that are capable of acetate oxidation in syntrophy with hydrogenotrophic methanogens (Ito et al., 2011) and thus may have been consuming some of the produced acetate. Hydrogenotrophic methanogens consume H_2 produced as a fermentation product to reduce its partial pressure, thus further driving fatty acid-producing reactions (Hattori, 2008). Amongst the fermenting community, members of the phylum Firmicutes were relatively abundant in the core community as well. These are generally anaerobic heterotrophs, fermenting amino acids and carbohydrates to produce volatile fatty acids (VFAs) (including acetate) and H_2. These included: *Sedimentibacter*, which are strictly anaerobic fermenters of pyruvate or amino acids to produce VFAs, but not H_2 (Breitenstein et al., 2002), *Proteiniborus* (Niu et al., 2008; Hahnke et al., 2018), *Lutispora*, which only ferment amino acids (Shiratori et al., 2008; Stackebrandt, 2014), and *Petrimonas,* which are capable of facultative aerobic growth (Grabowski et al., 2005; Hahnke et al., 2016). The genus *Gelria*, a strict anaerobic chemoorganotroph, grows on amino acids and sugars and is known to produce H_2 in the presence of a methanogenic partner. Depending on the available substrate, it can produce H_2, CO_2, and acetate (Plugge, 2015). Members of the phylum Chloroflexi are frequently reported in anaerobic digestor communities (Ariesyady et al., 2007; Yi et al., 2014; Liu et al., 2016; Petriglieri et al., 2018; Sun et al., 2016). The Bacteroidetes phylum includes proteolytic (degrading proteins to VFAs and ammonia; Yi et al., 2014) and fermentative bacteria. This phylum was represented in the enriched biocathodes by *Petrimonas* sp., a fermentative acidogenic bacteria that produces acetate, H_2, and CO_2 (Lu et al., 2012; Grabowski et al., 2005). They may also use acetate as their sole carbon source, with a possible indirect role in hydrocarbon metabolism (Elshahed et al., 2003) and have been found in MEC biofilms (Lu et al., 2010, Lu et al., 2012). The other genus detected was *vadinBC27 wastewater-sludge group*, an uncultured genus that has been described previously in anaerobic digestion (Liu et al., 2016).

References

1. Ariesyady, H.D., Ito, T., Okabe, S. (2007). Functional bacterial and archaeal community structures of major trophic groups in a full-scale anaerobic sludge digester. Water Res., 41, pp. 1554-1568
2. Breitenstein, A., Wiegel, J., Haertig, C., Weiss, N., Andreesen, J. R., & Lechner, U. (2002). Reclassification of Clostridium hydroxybenzoicum as Sedimentibacter hydroxybenzoicus gen. nov., comb. nov., and description of Sedimentibacter saalensis sp. nov.. International journal of systematic and evolutionary microbiology, 52(3), 801-807
3. Elshahed, M. S., Senko, J. M., Najar, F. Z., Kenton, S. M., Roe, B. A., Dewers, T. A., Spear, J. R. & Krumholz, L. R. (2003; Appl Environ Microbiol 69, 5609–562

4. Grabowski, A., Tindall, B. J., Bardin, V., Blanchet, D., & Jeanthon, C. (2005). - Petrimonas sulfuriphila gen. nov., sp. nov., a mesophilic fermentative bacterium isolated from a biodegraded oil reservoir.International Journal of Systematic and Evolutionary Microbiology, 55(3), 1113-1121
5. Hahnke, S., Langer, T., & Klocke, M. (2018). Proteiniborus indolifex sp. nov., isolated from a thermophilic industrial-scale biogas plant. International journal of systematic and evolutionary microbiology.
6. Hahnke, S., Langer, T., Koeck, D. E., & Klocke, M. (2016). Description of Proteiniphilum saccharofermentans sp. nov., Petrimonas mucosa sp. nov. and Fermentimonas caenicola gen. nov., sp. nov., isolated from mesophilic laboratory-scale biogas reactors, and emended description of the genus Proteiniphilum. International journal of systematic and evolutionary microbiology, 66(3), 1466-1475.
7. Hattori, S., 2008. Syntrophic acetate-oxidizing microbes in methanogenic environments. Microbes and Environments, 23(2), pp.118-127
8. Ito, T., Yoshiguchi, K., Ariesyady, H.D. and Okabe, S., 2011. Identification of a novel acetate-utilizing bacterium belonging to Synergistes group 4 in anaerobic digester sludge. The ISME journal, 5(12), p.1844.
9. Liu, C., Li, H., Zhang, Y., Si, D., & Chen, Q. (2016). Evolution of microbial community along with increasing solid concentration during high-solids anaerobic digestion of sewage sludge. Bioresource technology, 216, 87-94.
10. Lu, L., Xing, D., & Ren, N. (2012). Pyrosequencing reveals highly diverse microbial communities in microbial electrolysis cells involved in enhanced H2 production from waste activated sludge. Water research, 46(7), 2425-2434.,
11. Lu, L., Xing, D., Xie, T., Ren, N., & Logan, B. E. (2010). Hydrogen production from proteins via electrohydrogenesis in microbial electrolysis cells. Biosensors and Bioelectronics, 25(12), 2690-2695,
12. Niu, L., Song, L., & Dong, X. (2008). Proteiniborus ethanoligenes gen. nov., sp. nov., an anaerobic protein-utilizing bacterium. International journal of systematic and evolutionary microbiology, 58(1), 12-16.
13. Petriglieri, F., Nierychlo, M., Nielsen, P. H., & McIlroy, S. J. (2018). In situ visualisation of the abundant Chloroflexi populations in full-scale anaerobic digesters and the fate of immigrating species. PloS one, 13(11), e0206255.
14. Plugge, C.M., 2015. Gelria. Bergey's Manual of Systematics of Archaea and Bacteria, pp.1-2
15. Shiratori, H., Ohiwa, H., Ikeno, H., Ayame, S., Kataoka, N., Miya, A., ... & Ueda, K. (2008). Lutispora thermophila gen. nov., sp. nov., a thermophilic, spore-forming bacterium isolated from a thermophilic methanogenic bioreactor digesting municipal solid wastes. International journal of systematic and evolutionary microbiology, 58(4), 964-969.
16. Stackebrandt, E. (2014). The family Gracilibacteraceae and transfer of the genus Lutispora into Gracilibacteraceae. In The Prokaryotes (pp. 149-151). Springer, Berlin, Heidelberg.
17. Sun, L., Liu, T., Müller, B., & Schnürer, A. (2016). The microbial community structure in industrial biogas plants influences the degradation rate of straw and cellulose in batch tests. Biotechnology for biofuels, 9(1), 128.
18. Yi, J., Dong, B., Jin, J., Dai, X. (2014) Effect of increasing total solids contents on anaerobic digestion of food waste under mesophilic conditions: performance and microbial characteristics analysis. PLoS ONE, 9, p. e102548
